# Optic nerve astrocytoma in a dog

**DOI:** 10.1002/ccr3.612

**Published:** 2016-07-27

**Authors:** Orr Rozov, Pablo E. Piñeyro, Kurt L. Zimmerman, Ian P. Herring, Rachel Matusow, John H. Rossmeisl, Bernard S. Jortner, Jennifer Dreyfus

**Affiliations:** ^1^ Department of Biomedical Sciences and PathobiologyVirginia‐Maryland College of Veterinary MedicineVirginia TechBlacksburgVirginia24061‐0442USA; ^2^ Department of Veterinary Diagnostic and Production Animal MedicineCollege of Veterinary MedicineIowa State UniversityAmesIowa50011‐1250USA; ^3^ Department of Small Animal Clinical SciencesVirginia‐Maryland College of Veterinary MedicineVirginia TechBlacksburgVirginia24061‐0442USA; ^4^ Department of Pathobiological SciencesSchool of Veterinary MedicineUniversity of Wisconsin ‐ MadisonMadisonWisconsin53706USA

**Keywords:** Neurology, oncology, ophthalmology, veterinary

## Abstract

Intracranial astrocytomas are relatively uncommon in dogs and optic nerve astrocytomas even more so. This neoplasm should be considered as differential in canine patients with vision loss, retinal detachment, ocular mass, and histopathologic findings of infiltrative fusiform to polygonal glial cells possibly associated with glomeruloid vascular proliferation.

## Introduction

Intraocular neoplasms in dogs are uncommon in comparison with other anatomic locations 1, 2, 3, 4. Over 75% of these cases are attributed to melanocytic neoplasia 1. A majority of the remaining cases (~22%) are comprised of lymphoma, metastatic neoplasia, iridociliary epithelial tumors, optic nerve meningiomas, and histiocytic sarcomas in decreasing order of frequency 1, 2. Rarely, optic nerve astrocytomas have been reported in humans, dogs, and horses 5, 6, 7. In humans, astrocytomas involving the optic nerve are uncommon accounting for only about 1% of neoplasms at this site in comparison with 25% of neoplasms occurring in the brain 5, 8, 9, 10, 11, 12, 13, 14. Most of these neoplasms occur unilaterally, are benign, and arise in children under 10 years of age; those involving the cerebellum have a more favorable prognosis 8, 14. These younger patients commonly have an underlying familiar disorder such as neurofibromatosis. In older patients in their fourth and fifth decade of life, astrocytomas more commonly involve the cerebrum with a less favorable prognosis and can be associated with familiar disorders such as Li–Fraumeni syndrome 13, 15, 16, 17.

Most canine ocular astrocytoma cases are sporadic and not associated with any familial disorder 1, 13, 18, 19, 20, 21. However, Thomas et al. demonstrated a genomic risk factor associated with frequency of chromosome copy number aberrations within canine brain astrocytomas and tumor grade 22. Similar to humans, canine astrocytomas account for less than 1% of ocular and optic nerve neoplasms and 10–36% of primary intracranial neoplasms 1, 4, 16, 17, 18, 20, 21, 23. There appears to be a breed predisposition for development of intracranial astrocytomas in English Toy Spaniels, Boston Terriers, French Bulldogs, Boxers, and English Bulldog with a peak prevalence at 7–8 years and 1.5 odds ratio in favor of larger versus smaller breeds 13, 23. These risk factors have not been shown in association with canine ocular forms of this neoplasm 1, 16, 17. Gender as a risk factor has not been described for either anatomic location 1, 13, 16, 17, 18, 23.

Case reports related to ocular astrocytomas are still rare in veterinary literature. The purpose of this report is to add to this sparse body of information. This report presents the clinical, histological, and immunohistochemical features of an optic nerve astrocytoma in a dog.

## Case Report

An 11‐year‐old male boxer dog presented to the Virginia‐Maryland College of Veterinary Medicine Veterinary Teaching Hospital with a history of a unilateral nonhealing corneal ulcer involving the right eye. The dog had previously been treated for a spontaneous chronic corneal epithelial defect (SCCED) affecting the left cornea approximately 2 years prior to this visit.

Upon presentation, menace response, dazzle reflex, and direct pupillary light reflex in the right and consensual reflex to the left eye were absent. The right cornea exhibited mild diffuse edema and an approximately 5–6 mm superficial ulcer with loose adjacent corneal epithelium affecting the ventrolateral cornea. Blood‐tinged aqueous flare was present, hampering detailed anterior segment examination, but 360° posterior synechia was suspected. Examination of the lens and posterior segment was precluded by the anterior segment disease. Examination of the left eye was unremarkable, with the exception of faint ventrolateral corneal fibrosis and associated corneal ghost vessels at the site of the previous SCCED. Ocular ultrasound revealed complete retinal detachment and hyperechoic subretinal fluid. General physical examination was unremarkable and indirect blood pressure measurement was normal. Complete blood count and urinalysis were also unremarkable, while serum biochemistry profile^1^ revealed moderate elevations in serum ALT (624, 16–75 U/L), GGT (27, 1–5 U/L), and ALP (967, 8–70 U/L).

Abdominal ultrasound demonstrated enlargement of the right adrenal gland and atrophy of the left adrenal, suggestive of functional adrenal gland neoplasia.

Due to the patient's vision loss, concern for neoplastic disease, and risk of secondary glaucoma, enucleation was recommended. Following premedication with intramuscular acepromazine (0.02 mg/kg) and morphine (0.004 mg/kg), anesthesia was induced with an intravenous propofol bolus (2.6 mg/kg) and maintained with inhalant isoflurane. Perioperative cefazolin (22 mg/kg) was administered intravenously and a retrobulbar anesthetic block was performed utilizing bupivacaine (2 mL). Following routine clip and aseptic preparation of the right periocular area, the right globe and adjoining optic nerve were enucleated via a transconjunctival approach. The globe was placed in Davidson's fixative and submitted for histopathologic evaluation. Recovery from general anesthesia was uneventful and the dog was discharged the following day to the care of its owner with carprofen (1 mg/kg) for 10 days as needed for pain. The dog was clinically well at a 6 months’ post surgery phone recheck.

**Figure 1:**
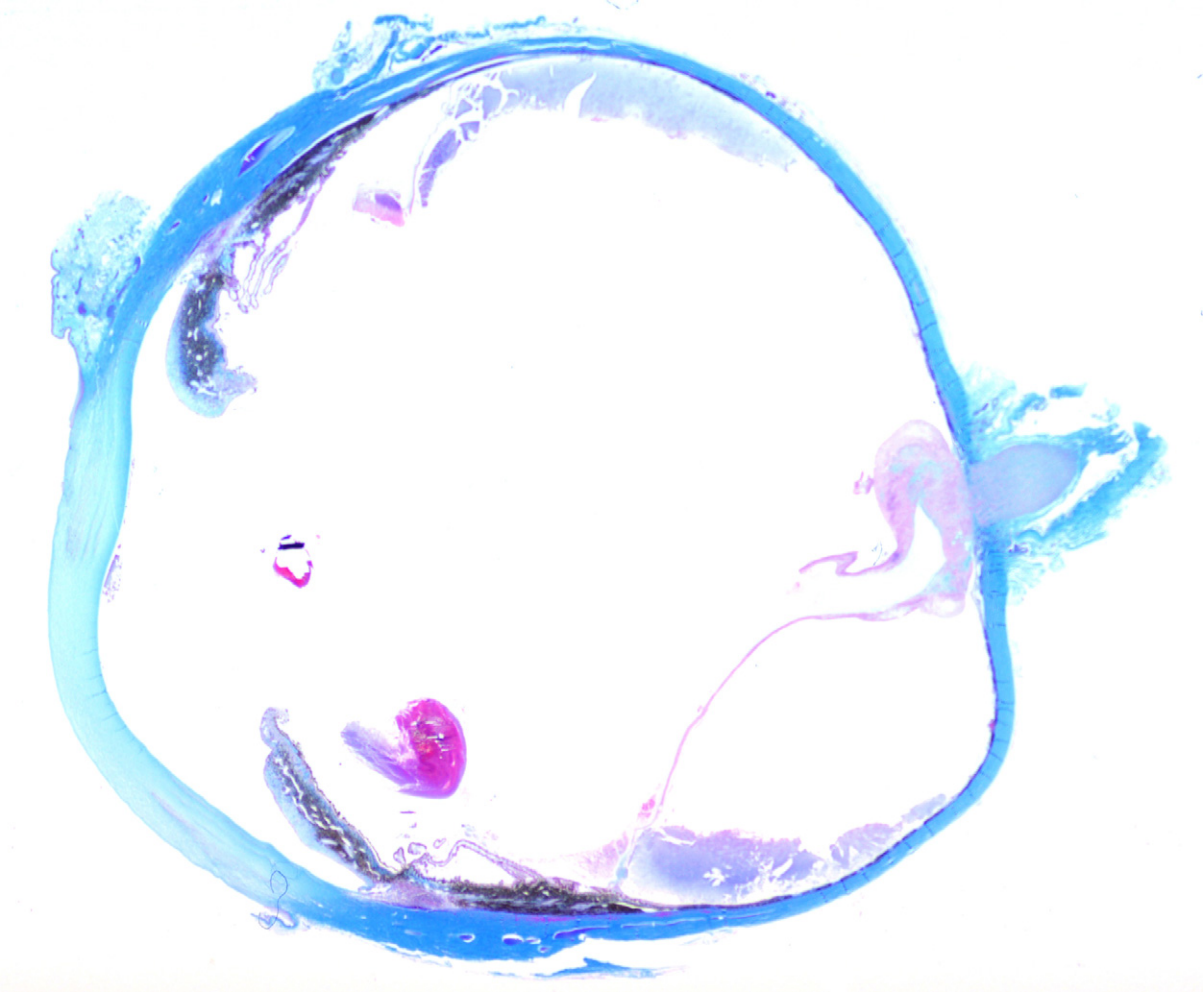
Subgross, sagittal section, left globe. Mass in area of optic papilla and detached retina. Trichrome.

**Figure 2:**
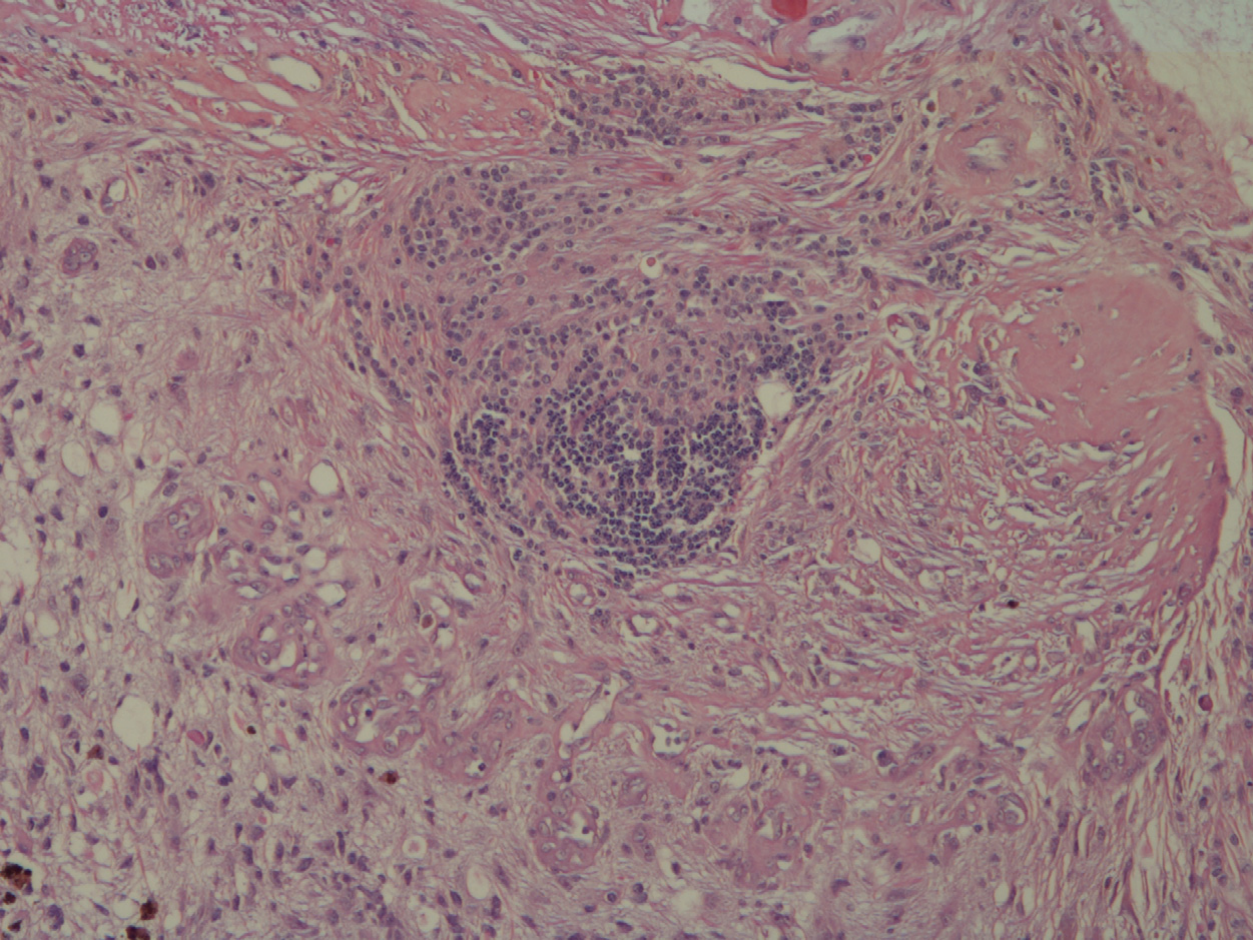
Left globe, optic papilla mass. Moderately cellular neoplasm arising from the optic cup composed of streaming spindle cells arranged in short stacks and bundles with areas of entrapped residual retinal nuclear cell layers and glomeruloid vascular proliferation. 100×, Hematoxylin and eosin.

**Figure 3:**
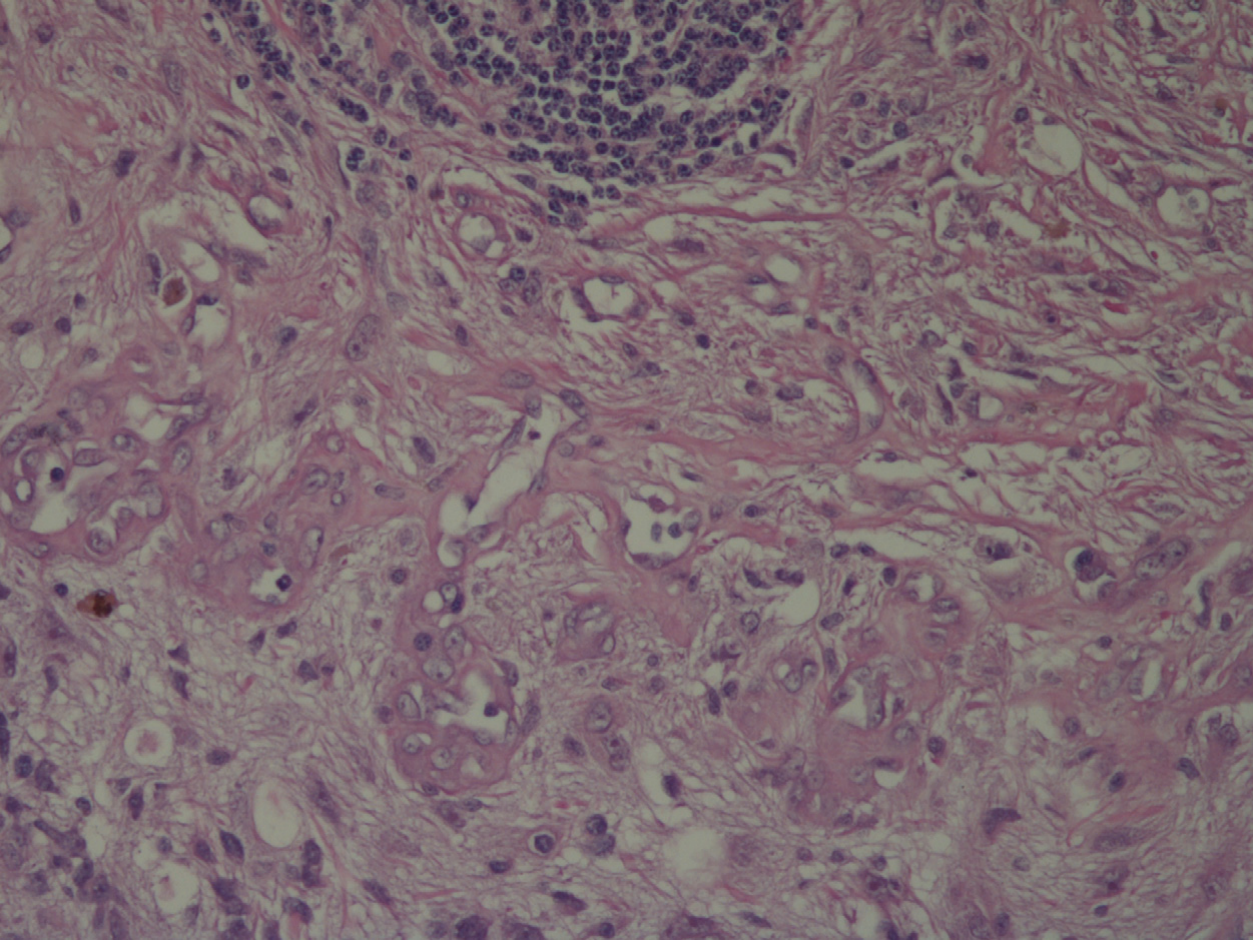
Left globe, optic papilla mass. Higher magnification of glomeruloid vascular proliferation and oval vesicular nuclei of adjoining neoplastic cells. 200×, Hematoxylin and eosin.

**Figure 4:**
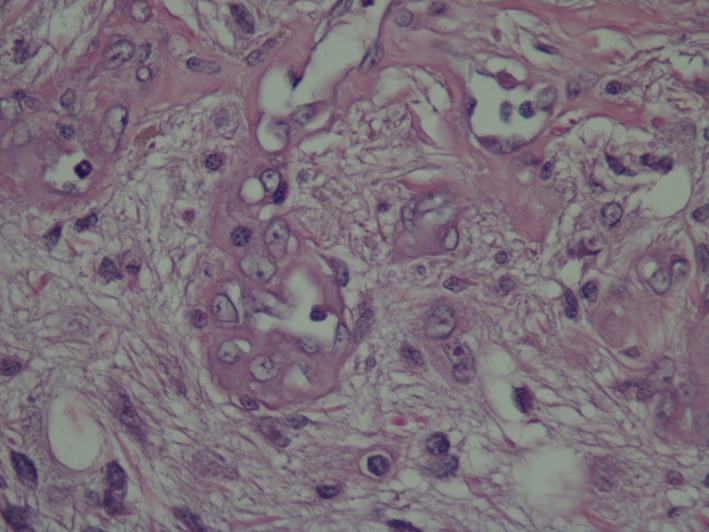
Left globe, optic papilla mass. Neoplastic cells have abundant pale fibular eosinophilic cytoplasm with indistinct borders, nuclei are open and vesicular with 1–2 nucleoli, and no mitotic figures are observed. 400×, Hematoxylin and eosin.

Histopathologic examination of a dorsal/ventral, transversion, section through the optic nerve and central cornea of the globe revealed attenuated corneal epithelium covered by homogenous eosinophilic proteinaceous debris. The corneal stroma was vascularized and expanded by edema and scattered macrophages and neutrophils. Both drainage angles appeared open. Focally, the ciliary body and iris were expanded by edema. The posterior chamber contained abundant pale eosinophilic fibular proteinaceous material (fibrin) mixed with a few neutrophils and macrophages. The lens was fractured in processing. The retina was detached throughout its length with loss of inner nuclear, plexiform, and ganglion layers; the outer nuclear layer was thinned and disarrayed. The pigmented retinal epithelium was hyperplastic. There was a moderately cellular, focally infiltrative mass arising from the junction of the optic papilla and optic nerve from which the retinal remnants arose (Fig. 1). The mass was composed of polygonal to stellate cells in interlacing bundles with intermixed solid areas of glomeruloid vascular proliferations supported by a fine fibrovascular stroma (Figs. 2 and 3). Neoplastic cells had oval nuclei 8–15 μm in length with 1.5× anisokaryosis, stippled chromatin, and 1–2 variably sized nucleoli (Fig. 4). Less than one mitosis was seen in three high‐power fields of view (10 fields not available for examination).

**Figure 5:**
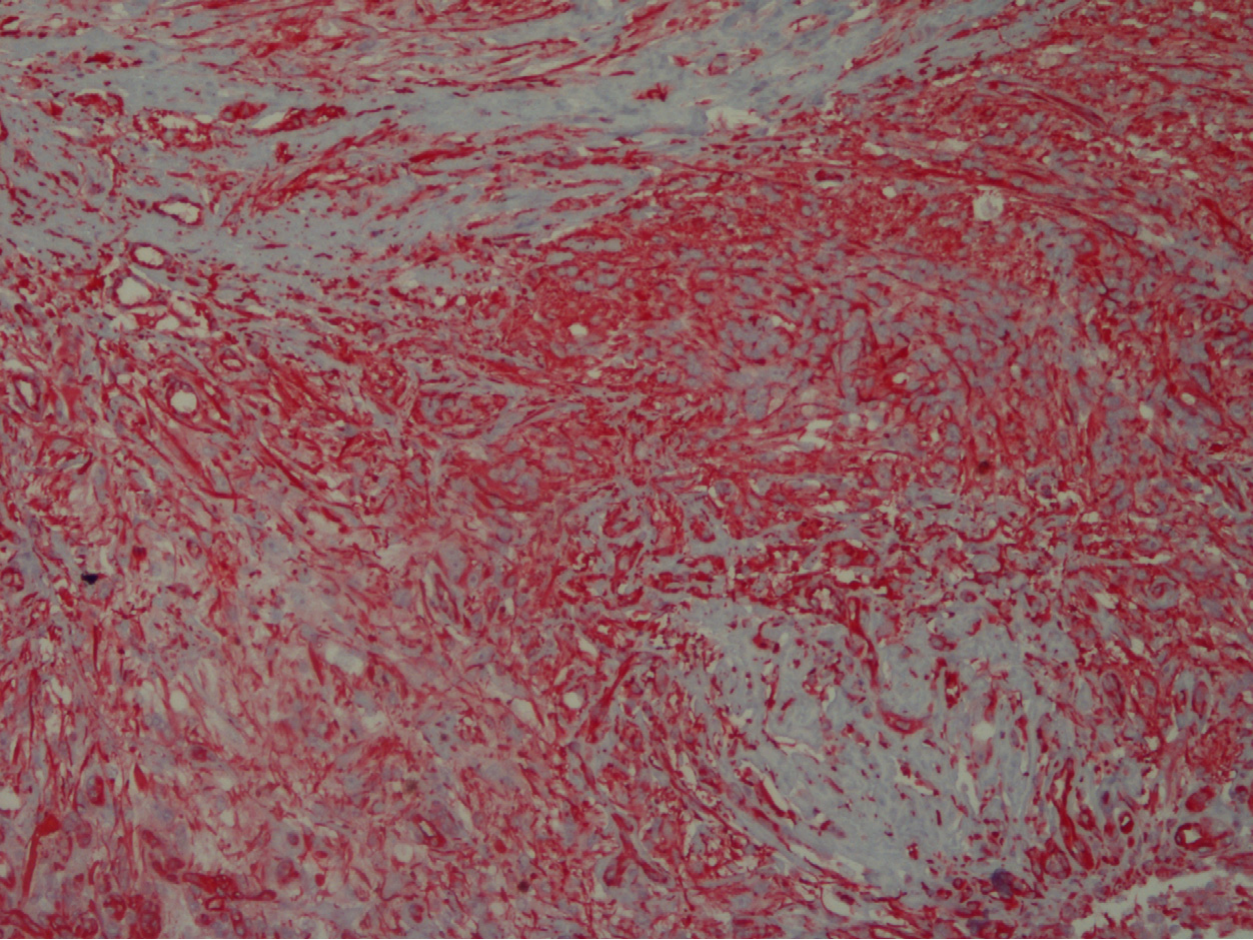
Left globe, optic cup mass. Moderate number of neoplastic cells have moderate positive (red) cytoplasmic vimentin immunoreactivity. 100×, vimentin, horseradish peroxidase.

**Figure 6:**
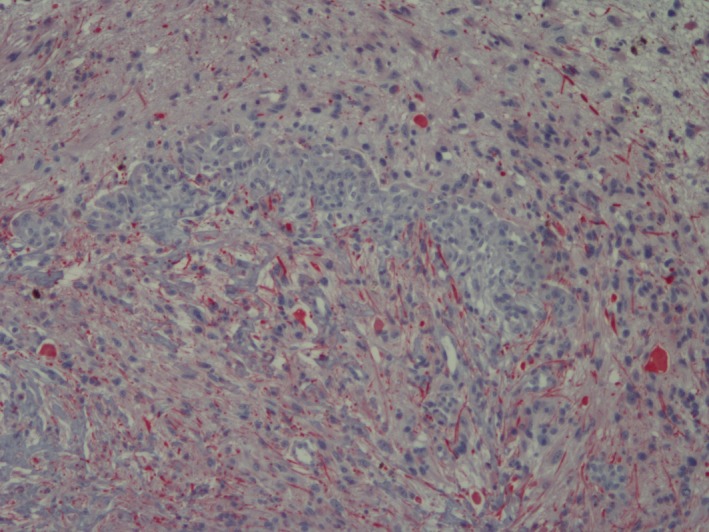
Left globe, optic cup mass. Majority of neoplastic cells have weak positive (red) cytoplasmic fibular process with GFAP immunoreactivity. 100×, GFAP, horseradish peroxidase.

**Figure 7:**
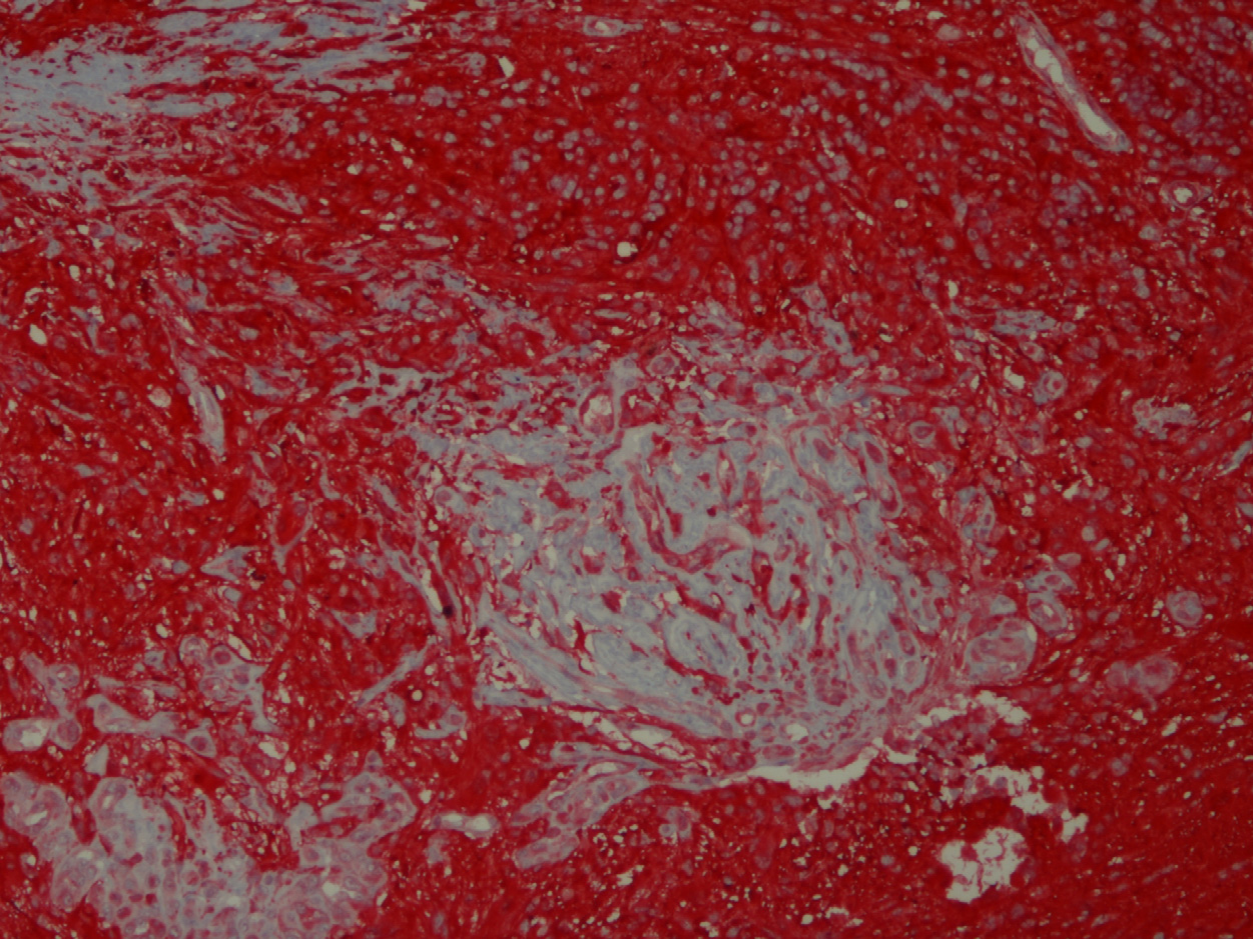
Left globe, optic cup mass. Majority of neoplastic cells have strong positive (red) cytoplasmic S100 immunoreactivity. 100×, S100, horseradish peroxidase.

Replicate sections with Masson's trichrome and luxol fast blue (LFB) histochemical stains, and vimentin, glial fibrillary acidic protein (GFAP), S100, and neuron‐specific enolase (NSE) immunohistochemical stains were examined. Trichrome staining indicated only scant amounts of collagen in mass. Approximately 50% of the neoplastic cells exhibited moderate cytoplasmic staining with vimentin (Fig. 5). A majority of the neoplastic cells had weak to moderate GFAP cytoplasmic immunoreactivity and strong S100 (Fig. 6 and 7). Neoplastic cells did not react with the LFB or NSE stains (images not shown). Negative and positive control slides reacted appropriate for these special staining procedures. Based on microscopic features and special stains results, the histopathologic diagnosis was optic nerve astrocytoma, grade II.

## Discussion

Gliomas are primary tumors of the central nervous system that are derived from glial cells 13, 18, 20. Types of gliomas include astrocytomas, oligodendrogliomas, and ependymomas. Astrocytomas most frequently occur in the cerebral hemispheres and are seen predominantly within the temporal‐piriform region; they may, however, be found in any part of the CNS including the brain stem, cerebellum, and spinal cord 13, 19, 20, 23, 24, 25. Though intracranial tumors are rare overall, of the intracranial neuroepithelial tumors, astrocytoma is the most common in domestic animals. Its highest incidence is in brachycephalic dogs between 8‐ and 9‐years of age and most commonly involves the cerebral hemispheres 13, 20. Many astrocytomas present as diffusely infiltrative growths whose margins are difficult to distinguish from adjoining neuropil making their complete surgical resection challenging 11, 12, 26, 27, 28.

Of the diffuse astrocytomas, fibrillary astrocytomas are most common, and are composed of diffusely infiltrating, elongate, spindyloid, and polygonal cells that appear as a mild increase in cellularity 13, 25. Neoplastic astrocytes typically demonstrate nuclear atypia with irregular hyperchromatic nuclei and varied cytoplasmic process appearance and fibrillarity. Diffusely infiltrative astrocytic tumors lacking mitotic figures are defined as grade II using the 2007 WHO schema 19. Anaplastic astrocytomas are composed of more densely cellular areas with greater pleomorphism than fibrillary astrocytomas. Often mitotically active cells are visible in the field. Anaplastic astrocytomas are classified as grade III via the WHO schema 19. Glioblastomas appear histologically similar to anaplastic astrocytomas, but they additionally include evidence of necrosis and vascular or endothelial proliferation (glomeruloid). Vascular proliferations, as seen in this case, appear as “tufts” of piled‐up endothelial cells that protrude into the lumen of vasculature. Astrocytic tumors with these features are classified as Grade IV using 2007 WHO grading criteria 13, 19, 20, 25. Grade I variants of this neoplasm are uncommon and are restricted to circumscribed pilocytic forms of the tumor. Prognostically in people, Grade II neoplasms are associated with survival of 5 years or more, 2–3 years for grade III, and <1 year for grade IV neoplasms 19.

For this case, the tumor's low mitotic rate, infiltration, and lack of necrosis are most compatible with a Grade II astrocytoma. However, the glomeruloid vascular proliferation suggests the neoplasm has acquired limited features associated with higher grade tumors 29.

Normal astrocytes are reliably identified by the presence of glial fibrillary acidic protein (GFAP), the main component astrocytic intermediate filaments. A large number of cells express GFAP in well‐differentiated astrocytomas 5, 7, 8, 12, 13, 16, 17, 19, 30, 31. In this case, the GFAP immunoreactivity of the astrocytes was weak, but this is not unusual for more anaplastic forms of this neoplasm 19, 25, 31. There is evidence that suggests an inverse proportionality between GFAP expression and the extent of anaplasia in astrocytic neoplasms as it may reflect the undifferentiated nature of the tumor cells [13].

Ocular astrocytomas in dogs are very rare with only a few reported cases 1, 2, 4, 13, 21. Ocular astrocytomas can appear clinically as discrete masses in the fundus and may be misdiagnosed as nonneoplastic lesions 15, 31, 32, 33. Hyphema is the most common presenting clinical finding accompanied, in decreasing order of frequently, with evidence of a fundic mass, retinal detachment, blindness, and exophthalmia (Review). Microscopically, high‐grade astrocytomas are more commonly associated with glomeruloid vascular proliferation, as seen in this case. The few other reported optic nerve and retina astrocytomas in the literature shared many of the morphologic and histologic features as seen in those arising in the brain 1, 6, 7, 16, 17, 21, 30, 34, 35.

Further diagnostics related to the enlarged adrenal gland were not pursued in this patient at the decision of the owner. The dog did not have any clinical signs of hyperadrenocorticism at its time of presentation and imaging results were not typical of a pheochromocytoma. It is possible the adrenal enlargement was associated with a cortical hyperplasia, adenoma, metastatic disease, or other, but this remains speculative without further pituitary–adrenal axis functional testing. The adrenal change was not thought to have any association with the ocular neoplasm in this case.

This case report adds to the existing small body of literature dealing with ocular astrocytomas in companion animals. It also serves as a reminder for the veterinary ophthalmologist and pathologist that, although they are rare, retinal and optic nerve gliomas should be included as differential diagnoses for intraocular and orbital masses.

## Conflict of Interest

None declared.

## Note

1 AU480 ‐ Beckman Coulter, Inc., Brea CA; Virginia Tech Animal Laboratory Services, Blacksburg, VA.
